# Transient cardiac dysfunction but elevated cardiac and kidney biomarkers 24 h following an ultra-distance running event in Mexican Tarahumara

**DOI:** 10.1186/s13728-017-0057-5

**Published:** 2017-12-11

**Authors:** Dirk L. Christensen, Diana Espino, Rocío Infante-Ramírez, Mónica S. Cervantes-Borunda, Rosa P. Hernández-Torres, Antonio E. Rivera-Cisneros, Daniel Castillo, Kate Westgate, Dijana Terzic, Soren Brage, Christian Hassager, Jens P. Goetze, Jesper Kjaergaard

**Affiliations:** 1 Global Health Section, University of Copenhagen, Øster Farimagsgade 5, building 9, 1014 Copenhagen K, Denmark; 2 MRC Epidemiology Unit, University of Cambridge, Cambridge, UK; 3 Faculty of Physical Education and Sport Sciences, Autonomous University of Chihuahua, Chihuahua, Mexico; 4 Faculty of Chemical Sciences, Autonomous University of Chihuahua, Chihuahua, Mexico; 5 Faculty of Nursing and Nutrition, Autonomous University of Chihuahua, Chihuahua, Mexico; 6 Faculty of Medicine, Autonomous University of Guadalajara, Jalisco, Mexico; 7 Faculty of Medicine, Autonomous University of Chihuahua, Chihuahua, Mexico; 8 Department of Clinical Biochemistry, Centre of Diagnostics, Copenhagen University Hospital, Rigshospitalet, Denmark; 9 Department of Cardiology, Heart Centre, Copenhagen University Hospital, Rigshospitalet, Denmark

**Keywords:** Echocardiography, Cardiac biomarkers, Kidney biomarkers, Ultra-marathon, Tarahumara

## Abstract

**Background:**

The Mexican Tarahumara are accustomed to running ultra-distance races. No data exist on the acute physiological changes following ultra-distance running and physiological-biomarker associations in this population. Thus, we aimed to investigate the acute impact (≤ 24 h) on functional and biochemical changes of the cardiac muscle and biochemical changes associated with kidney function following a 63-km ultra-distance race with an altitude difference of 1800 m in Mexican Tarahumara athletes.

**Methods:**

Ten Tarahumara male athletes (mean ± SD age = 29.9 ± 6.6 years) volunteered to participate in the study. VO_2_max was assessed by a sub-maximal step test individually calibrated combining heart rate and accelerometry. Standard transthoracic echocardiography methodology and venipuncture blood tests were carried out at four time points: pre-race, immediately post-race, 6 h, and 24 h post-race.

**Results:**

Estimated mean VO_2_max was 54.5 (± 8.8) mL O_2_ min^−1^ kg^−1^ and average physiological activity intensity was 746 (± 143) J min^−1^ kg ^−1^ (~ 11.5 METs). When compared to pre-race values, significant changes in left ventricular ejection fraction (LVEF) and LV end-diastolic volume (− 15%, *p* < 0.001 for both parameters), cardiac output (39%, *p* < 0.001), and maximal longitudinal velocity (− 13%, *p* < 0.009) were seen post-race with LVEF also being decreased at < 6 h post-race (− 8%, *p* < 0.014). Plasma biomarkers mid-regional pro-atrial natriuretic peptide, copeptin-ultra sensitive, and high-sensitivity cardiac troponin T remained significantly elevated at 24 h post-race, and the two latter were inversely associated with LVEF (*p* < 0.04). Kidney dysfunction was indicated by increased post-race copeptin-ultra sensitive.

**Conclusions:**

The athletes participating in this study had acute transient cardiac dysfunction as assessed by echocardiography but elevated cardiac and kidney biomarkers at 24 h following a 63-km race with extreme altitude variation.

**Electronic supplementary material:**

The online version of this article (10.1186/s13728-017-0057-5) contains supplementary material, which is available to authorized users.

## Background

The increasing popularity of ultra-endurance sports participation has fuelled an ever growing number of reports on physiological and biochemical cardiac and kidney dysfunction in elite as well as recreational athletes [1–3]. It has been established that engaging in extreme physical activities at altitude on a regular basis will result in acute changes of heart function at altitude [4]. When looking at the heart muscle, the focus has gradually shifted from primarily focusing on left ventricular (LV) dysfunction and abnormality [5] to also including right ventricular (RV) dysfunction and structural remodeling [2]. While LV remodeling as a result of regular endurance training seems to be a marker of positive health outcomes rather than cardiomyopathy, there is growing evidence that RV function may be more profoundly affected by vigorous endurance exercise [2].

Similarly, numerous studies have investigated changes in biomarkers associated with cardiac and kidney dysfunction as a consequence of ultra-endurance sports participation. These have included novel biomarkers such as mid-regional pro-atrial natriuretic peptide (MR-proANP) and copeptin-ultra sensitive (copeptin-us) primarily associated with myocardial pressure overload and wall-stretch as well as kidney damage/stress and hypoxia, respectively [6, 7]. Furthermore, several studies have used more conventional biomarkers such as high-sensitivity cardiac troponin T (hs-cTnT) release primarily associated with hypoxia-induced myocardial damage [8, 9]. The exercise-induced release of these biomarkers has been intensely discussed in the scientific literature with interpretations ranging from being a consequence of benign leakage from the cardiac myocyte membrane to being myocyte necrosis [10]. There is some evidence that aerobic exercise improves renal function due to different mechanisms such as inhibiting the renin–angiotensin–aldosterone system or improving renal blood flow through modification of dyslipidemia and intra-glomerular pressure [11–13]. However, intensive endurance exercise may result in rhabdomyolysis-induced acute renal dysfunction due to glomerulus degeneration and reduced renal blood flow leading to a reduced supply of oxygen and energy, resulting in ischemic vascular endothelial cell damage [14]. Previous reports covering physiological and biochemical changes of the heart and the kidneys have ranged from the marathon (42.2 km) to ultra-distance races of up to 218.5 km [1, 3, 15], and the average intensities have ranged from 7.0 to 9.9 km h^−1^. However, except for one recent investigation focusing on cardiac and kidney biomarkers [7] following ultra-distance running at moderate altitude in flat terrain in Mexican Tarahumara runners, none of these studies have reported results from indigenous American athletes.

For more than a century, the Tarahumara who reside in northern Mexico have been known for their prowess of ultra-distance running—here defined as distances longer than the marathon race (42.2 km)—in the *Sierra Madre Occidental* mountains (also known as the *Sierra Tarahumara*) with steep canyons deeper than 1800 m [16]. Early studies have reported moderate running intensity in the Tarahumara when engaging in long-distance running [17, 18]. More recently, we reported high daily physical activity in rural Tarahumara [19] indicating generally high activity levels in this population which may be introduced during childhood and persist throughout life. Thus, we aimed to study the combined acute impact (≤ 24 h) on and association between physiological and biochemical changes of the cardiac muscle following a 63-km ultra-distance race with an altitude difference of 1800 meters in Tarahumara athletes who have life-long adaptation to long-distance running and physical activity in mountainous terrain.

## Methods

Ten adult Tarahumara men from the village of Choguita, Chihuahua, who participated in a 63-km race of the *Ultramaratón de los Cañones* in the state of Chihuahua, Mexico, volunteered to participate in the study. The number of included participants was based on availability of subjects participating in the 63-km race at the time of the study. The event took place in July 2012, and began at 6:00 a.m. in the town of Guachochi at an altitude of ~ 2400 m; the route was a loop including a drop of ~ 1800 m into the bottom of the *Barranca de la Sinforosa* from where the runners had to return. All pre-race measurements were carried out on the day before the race. Through a contact person, the participants were instructed not to do strenuous exercise 3 days prior to the pre-race measurements, as well as abstain from smoking, alcohol consumption, and drinks containing caffeine on the day of the pre-race measurements as well as during the 24-h post-race period. Height and weight were measured to the nearest 0.1 cm and 0.1 kg, respectively, with the participant wearing minimal clothing, and body mass index (weight in kg/height in m^2^) was derived.

Systolic and diastolic blood pressures were measured using the auscultatory method (VSG-3 model, Phillips, Eindhoven, the Netherlands). Blood pressure was measured three times on the right upper arm with the participant seated and following 15 min of rest with 2 min in-between each measurement. Mean blood pressure was calculated from the last two measurements. Elevated blood pressure was defined as systolic blood pressure ≥ 140 mm Hg and/or diastolic blood pressure ≥ 90 mm Hg [20].

Glycated hemoglobin (HbA1c) and hemoglobin (Hb) levels were determined by capillary blood. HbA1c was analyzed using an automated boronate affinity assay which minimizes interference from other Hb variants and derivates analyzed by an Afinion AS100 Analyzer (Alere-Axis Shield PoC, Oslo, Norway). Hb was analyzed by a modified azide-methaemoglobin reaction [21] using a Hb 2011 device (HemoCue AB, Ängelholm, Sweden). Anemia was defined as blood Hb below 13 mg/dL.

VO_2_max and race energy expenditure were estimated using a combined uniaxial accelerometer and heart rate (HR) sensor (Actiheart, CamNtech Ltd, Cambridge, UK) attached to the chest on two ECG electrodes (Unomedical A/S, Birkerød, Denmark). One day prior to the race, each participant performed an 8-min step test, stepping up and down to a voice prompt on a 21.5-cm-high step (Reebok, Lancaster, UK) using a sub-maximal ramped protocol [22]. The monitor was then initialized to record at 15-s intervals and participants wore the monitor continuously for the following 24 h (including the race period). For further details of the step test and physical activity monitoring, see [7].

Following the race, each participant did a quantitative physical activity and competition personal history in Additional file 1: Questionnaire (in Spanish), specifically prepared for the study.

A standard 12-lead electrocardiography (ECG) was performed using a Medi Trace, ver. 5.2 (Medi Core, Mexico City) pre- and post-race. The ECG was read by an experienced cardiologist, blinded to participant demographic and race data.

A standard transthoracic echocardiography was performed at pre-race (baseline), 30 min post-race, 6 h post-race, and 24 h post-race using at Philips^®^ Cx50 Ultrasound system, equipped with the S5-1 transducer. The patient was placed in the left lateral decubitus position and at least three cycles were recorded triggered to the ECG at end-expiration. Analysis of the recorded imaging was performed blinded to exercise efforts using the Philips Excelera version R3.3L1 and Qlab software version 9.1, applying the QMC solution for deformation analysis. RV and LV chamber dimensions were measured according the current guidelines [23]. LV mass was calculated according to the formula: 0.8*(1.04*[{LV internal dimension-diastole + posterior wall thickness at end diastole + superior wall thickness at end diastole)^3^ − (LV internal dimension-diastole)^3^]}, and then indexed to body surface area. Cardiac deformation was assessed from tracing the LV wall and septum in the apical 4-, 2-chamber and long-axis views in 17 segments [23]. The global longitudinal strain of the RV free wall was calculated as the mean of the basal, mid, apical, and apex segments in the apical 4-chamber view as recommended [23].

Ten mL blood was collected at each time point: pre-race (baseline), < 5 min post-race, 6 h post-race, and 24 h post-race. The blood samples were drawn by standard venipuncture into BD-Vacutainer^®^ tubes containing lithium heparin. All the samples were centrifuged, plasma transferred to cryogenic tubes, and frozen in aliquots at − 80 °C until transported on dry ice to Copenhagen University Hospital (Rigshospitalet), Denmark for analyses.

Plasma concentrations of hs-cTnT, creatine kinase (CK), and CK isoform myocardial band (MB), creatinine, high-sensitivity C-reactive protein, iron, haptoglobin, lactate dehydrogenase (LDH), albumin, sodium, and potassium were measured using the fully automated Roche/Hitachi MODULAR SYSTEMS platform (Roche Diagnostics GmbH, Mannheim, Germany); all analyses were accredited by the national accreditation body in Denmark (DANAK). MR-proANP and copeptin-us concentrations were measured using the Kryptor Compact Plus platform (Thermo Fisher Scientific, BRAHMS, Hennigsdorf, Germany); the validation of the two analyses has been reported previously [24, 25].

### Statistics

All background and descriptive results are given as mean (SD). Within and between subject variation of echocardiographic measurements, plasma markers and ECG were calculated using linear regression analyses to test for associations between exposure and outcome variables as well as between different outcome variables. Age was used as explanatory variable, and in all linear regression analyses the pre-race (baseline) results were used as references. Multivariate regression analyses were performed in order to rule out the possibility that pre-race LVEF level confounded the relationship between LVEF and VO_2_max and in order to determine if the post-race LVEF results were explained by high or low VO_2_max (study participants were allocated to two groups of five each, under and above the median of 52.5 mL O_2_ min^−1^ kg^−1^, respectively). The latter multi-regression analysis tested an interaction between time of post-race LVEF levels and VO_2_max group including pre-race LVEF in the analysis as well. Plasma marker results have been adjusted for albumin as a proxy of hydration in order to present hydration-independent results. In case of participants having plasma values below the detection limit for a plasma marker, data have been presented as median (IQR). A value between 0 and the detection limit was randomly chosen and used in the regression analysis. After the first post-race measurements, two of the ten study participants failed to show up for the last two post-race measurements. As a result, all analyses have been carried out based on ten participants for the first two measurements, and based on eight participants for the last two measurements. *p* < 0.05 was considered statistically significant. All analyses we done with Stata 13.1 Intercooled version (Stata Corp, College Station, TX).

## Results

Mean age of the subjects was 29.9 (± 6.6) years, ranging from 22 to 45 years of age. No abnormalities, i.e., valve disease or non-training-related ECG changes were observed during the pre-race echocardiographic and ECG tests with small, statistically non-significant fluctuations of ST segment elevation or depression post-race (results not shown).

Background characteristics are presented in Table 1. Estimated mean VO_2_max was 54.5 (± 8.8) mL O_2_ min^−1^ kg^−1^, with the average physiological (activity) intensity during the race being 746 (± 143) J min^−1^ kg^−1^ (~ 11.5 METs including resting energy expenditure) or if expressed relative to individual VO_2_max approximately 72 (± 6) % of estimated VO_2_max. Mean duration of race time was 418.3 (± 42.5) min, and mean absolute intensity was 9.1 (± 0.9) km h^−1^. Estimated VO_2_max, HR measures, and absolute and relative intensities of the 63-km race are presented in (Table 2).

**Table 1:** Background characteristics of male Tarahumara runners (*n* = 10) presented as mean (SD)

Variable	Mean	SD	Range
Age (years)	29.9	6.6	22–45
Height (cm)	162	0.1	155–177
Weight (kg)	54.2	5.7	46.0–64.6
Body mass index (kg/m^2^)	21.2	1.7	18.7–24.1
Waist circumference (cm)	76.5	4.5	68.2–83.4
Body fat (%)^a^	10.6	1.5	8.0–12.7
Body surface area (m^2^)	1.22	0.15	1.00–1.47
Systolic blood pressure (mmHg)^b^	105	9	96–126
Diastolic blood pressure (mmHg)^2^	64	7	56–78
Glycated hemoglobin (%)	5.7	0.2	5.3–5.9
Hemoglobin (g/dL)	15.7	1.5	12.4–18.0
Hematocrit (%)	47.2	4.6	37.2–54.0

^a^ *n* = 9

^b^Blood pressure measured with subject in sitting position ≥ 15 min of rest

**Table 2:** Estimated VO_2_max, heart rate measures, absolute and relative intensities of 63-km running, and history of running competition in Tarahumara athletes (*n* = 10) presented as mean (SD)

Variable	Mean	SD	Range
Sleeping heart rate (bts min^−1^)	51	3	47–58
Estimated maximal heart rate (bts min^−1^)^a^	187	4	177–193
Estimated VO_2_max (mlO_2_ min^−1^ kg^−1^)	54.5	8.8	40.9–70.1
Race time (min)	418.3	42.5	351–485
Absolute intensity (km h^−1^)	9.1	0.9	7.8–10.8
Net heart rate during race (bts min^−1^)^b^	103	8.0	90–116
Physiological intensity (J min^−1^ kg^−1^)^c^	746	143	500–1002
Relative intensity (%)	72	6	61–79
Running competitions (n)*	8		0–25

* Number of running competitions within last year with distance/time ranging from 42 to 63 km to 24-h traditional kick-ball race estimated at 150–200 km (based on answers from 8 participants). One participant reported having experienced hematuria

^a^Estimated maximal heart rate according to Tanaka equation

^b^Net heart rate is heart rate above sleeping level

^c^Activity intensity during race without resting energy expenditure

**Fig. 1:**
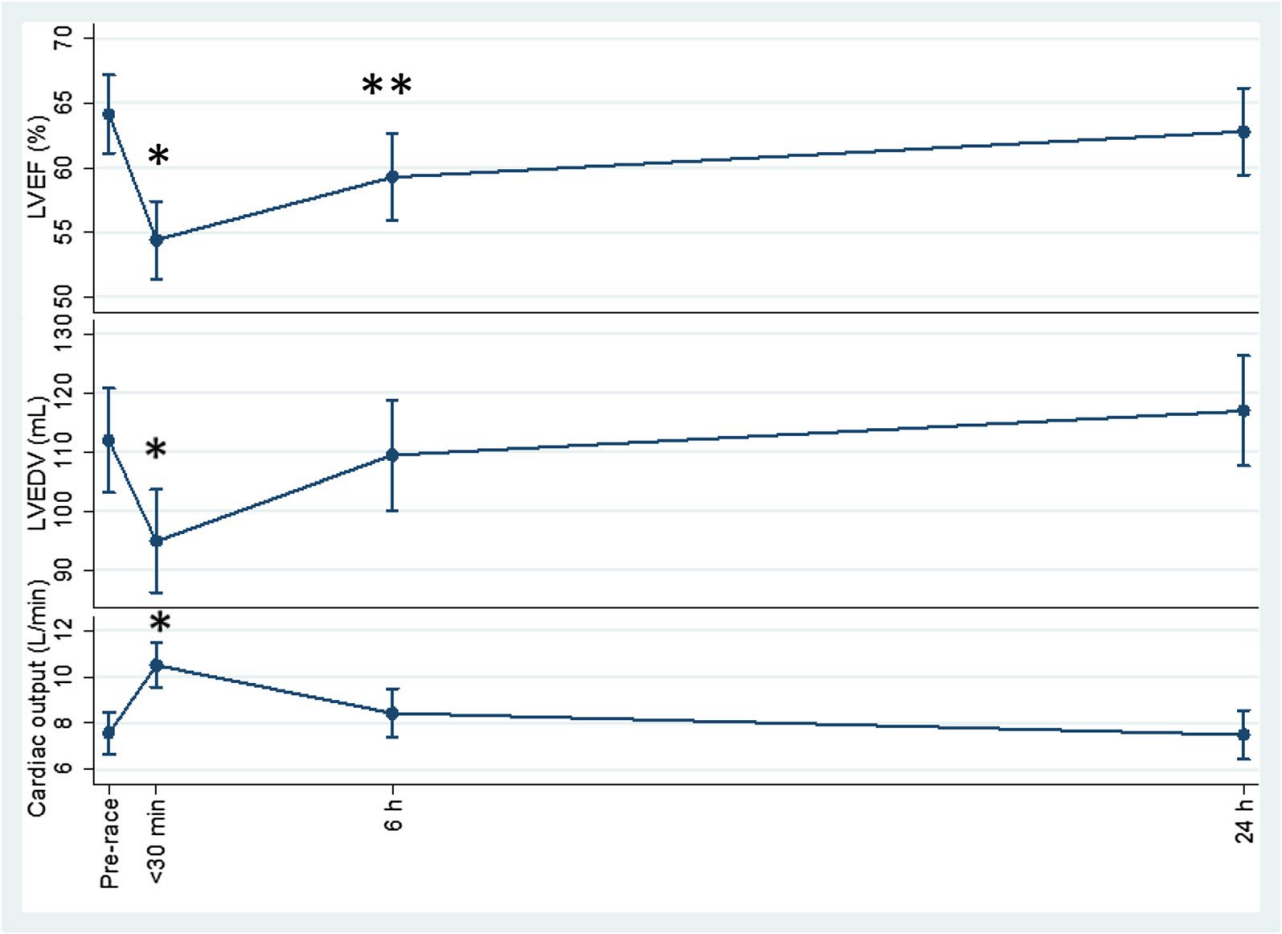
Within subject variation of left ventricular ejection fraction (%), left ventricular end-diastolic volume (mL), and cardiac output (L min^−1^) with age as explanatory variable, using the baseline values as the references was done by repeated measurements (mean (95% CI)) at time points 30 min, 6 h, and 24 h after running 63 km. *Denotes *p* < 0.01; **Denotes *p* < 0.05

**Fig. 2:**
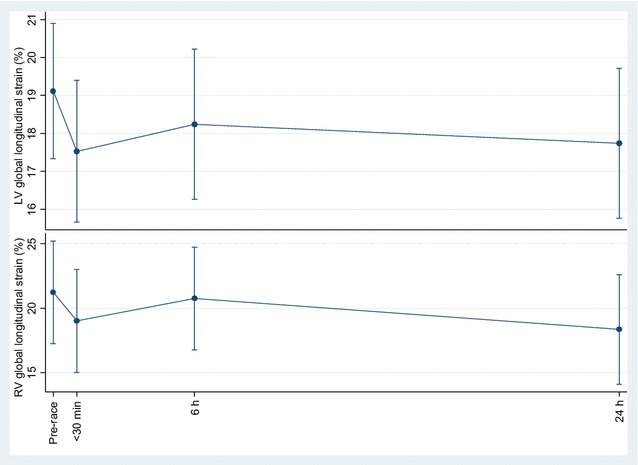
Within subject variation of left ventricular global longitudinal strain (%), and right ventricular global longitudinal strain (%) with age as explanatory variable, using the baseline values as the references was done by repeated measurements (mean (95% CI)) at time points 30 min, 6 h, and 24 h after running 63 km

Pre-race mean relative LV mass was 106 g m^−2^ (± 23), with all participants being within the reference range of 49–115 g m^−2^ according to [23] except for one, who had a mildly increased LV mass at 122 g m^−2^. Mean LV ejection fraction (EF), LV end-diastolic volume, and cardiac output showed the most marked changes compared to the pre-race values with all 30 min post-race changes (*p* < 0.001), and for the former 6 h post-race change also being significantly different (*p* < 0.014) (Fig. 1). Mean post-race cardiac deformation measurements LV global longitudinal strain (GLS) and estimated RV GLS did not show any significant changes compared to mean pre-race values (Fig. 2). Other echocardiographic measurements are shown in (Table 3) with post-race reduction of maximal longitudinal velocity (RV S’) (30 min, *p* < 0.009) and LV velocity time integral (30 min, *p* < 0.007 and 6 h, *p* < 0.041) and subsequently normalized. Post-race HR increased (30 min, *p* < 0.001 and 6 h, *p* < 0.002) and stroke volume decreased (30 min, *p* < 0.001 and 6 h, *p* = 0.057).

**Table 3:** Alterations of echocardiographic measures, plasma markers, and electrocardiogram following 63 km of running at moderate-to-low altitude in male Tarahumara (*n* = 10) presented as mean (95% CI)

Parameter	Pre-race	Post-race changes (mean values (95% CI) with statistical differences from pre-race value)					
		< 5-min/30-min	*p* value	6-h	*p* value	24-h	*p* value
Echocardiography^a,b^							
TAPSE (cm)	2.6 (2.4; 2.9)	− 0.2 (− 0.5; 0.1)	0.257	− 0.1 (− 0.4; 0.2)	0.654	0.4 (0.1; 0.7)	0.007
RV S’ (cm s^−1^)	15.5 (14.3; 16.7)	− 2.1 (− 3.6; − 0.5)	0.009	1.0 (− 0.6; 2.6)	0.233	0.8 (− 0.9; 2.5)	0.330
LV outflow VTI (cm)	22.1 (20.3; 23.8)	− 2.6 (− 4.6; − 0.7)	0.007	− 2.2 (− 4.3; − 0.1)	0.041	− 0.4 (− 2.5; 1.7)	0.699
LV SAX GCS (%)	17.8 (15.0; 20.6)	− 1.7 (− 5.1; 1.6)	0.303	2.5 (− 1.4; 6.3)	0.206	4.3 (0.7; 7.9)	0.019
Plasma biomarkers^a,c^							
LDH (U L^−1^)	102.8 (19.3; 186.3)	259.9 (176.4; 343.4)	0.004	209.4 (116.6; 302.1)	0.065	270.3 (177.5; 363.0)	0.004
Creatine kinase (U L^−1^)	140.0 (66.2; 213.8)	2737 (2683; 7267)	< 0.001	5535 (2298; 8772)	< 0.001	5380 (2234; 8527)	< 0.001
Creatine kinase-MB (µg L^−1^)	3.1 (1.3; 4.8)	62.5 (26.5; 98.6)	< 0.001	70.6 (27.0; 114.3)	< 0.001	56.3 (21.5; 91.0)	< 0.001
Creatine kinase ratio	2.3 (1.3; 3.3)	0.4 (− 1.0; 1.8)	0.573	− 0.9 (− 2.3; 0.6)	0.258	− 1.0 (− 2.5; 0.5)	0.179
Creatinine (µmol L^−1^)	64.9 (50.0; 79.8)	88.1 (73.2; 103.0)	0.002	88.6 (72.5; 104.7)	0.004	69.6 (53.5; 85.7)	0.565
hs-CRP (mg L^−1^)	0.8 (0.1; 1.4)	1.2 (0.3; 2.1)	0.336	9.9 (1.7; 18.1)	< 0.001	15.8 (2.8; 28.8)	< 0.001
Iron (µmol L^−1^)	15.1 (12.3; 17.9)	13.5 (11.7; 15.2)	0.226	7.0 (5.4; 8.6)	< 0.001	20.5 (17.4; 23.6)	0.004
Haptoglobin (g L^−1^)	0.9 (0.7; 1.1)	0.3 (0.2; 0.5)	<0.001	0.4 (0.2; 0.6)	< 0.001	0.8 (0.6; 1.0)	0.165
Sodium (mmol L^−1^)	138 (131; 146)	128 (121; 135)	0.020	141 (133; 149)	0.549	152 (144; 160)	0.005
Potassium (mmol L^−1^)	4.3 (3.8; 4.7)	4.6 (4.1; 5.0)	0.354	4.3 (3.8; 4.8)	0.917	4.5 (4.0; 5.0)	0.572
Albumin (g L^−1^)	46.8 (44.7; 48.8)	51.4 (49.3; 53.4)	< 0.001	47.0 (44.7; 49.2)	0.877	44.1 (41.8; ; 46.3)	0.045
Electrocardiogram^a,d^							
PQ interval (ms)	154 (147; 162)	− 4 (− 12; 3)	0.254			0.0 (− 8; 8)	0.975
QRS complex (ms)	86 (81; 91)	− 2 (− 6; 1)	0.230			− 2 (− 6; 1)	0.192
QTc (ms)	405 (390; 419)	19 (− 2; 40)	0.073			28 (6; 49)	0.011
Hemodynamics^a,d^							
Heart rate (bts min^−1^)	56 (51; 61)	33 (26; 40)	< 0.001	11 (4; 18)	0.002	4 (− 3; 11)	0.302
Stroke volume (L min^−1^)	7.1 (6.6; 7.7)	− 2.0 (− 2.6; − 1.3)	< 0.001	− 0.7 (− 1.3; 0.2)	0.057	1.7 (− 0.5,0.9)	0.612
SBP (mmHg)	105 (99; 111)	− 6 (− 13; 19	0.008	− 7 (− 15; 0.0)	0.043	− 8 (− 15; − 1)	0.030
DBP (mmHg)	64 (60; 67)	1 (− 3; 6)	0.613	− 6 (− 11; − 1)	0.018	− 9 (− 14; − 4)	< 0.001

*TAPSE* tricuspid annular plane systolic excursion; *RV S’* right ventricular annular maximal longitudinal velocity; *LV outflow VTI* left ventricular velocity time integral; *LV SAX GCS* left ventricular parasternal short-axis global circumferential strain; *LDH* lactate dehydrogenase; *creatine kinase-MB* creatine kinase isoform myocardial band; *hs-CRP* high-sensitivity C-reactive protein; *SBP* systolic blood pressure; *DBP* diastolic blood pressure

^a^Analyses age-adjusted

^b^First post-race echocardiographic measurement carried out 30 min post-race

^c^First post-race blood samples collected < 5 min post-race

^d^First post-race electrocardiogram measurement carried out < 40 min post-race

**Fig. 3:**
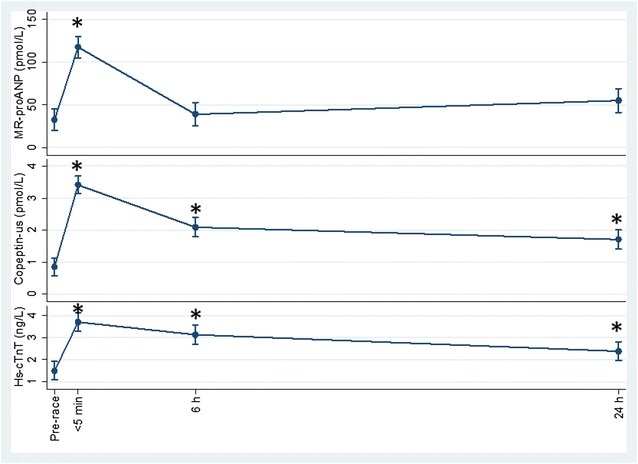
Within subject variation of mid-regional pro-atrial natriuretic peptide (pmol L^−1^), copeptin-ultra sensitive (pmol L^−1^), and high-sensitivity cardiac troponin T (ng L^−1^) with age as explanatory variable, using the baseline values as the references was done by repeated measurements (mean (95% CI)) at time points < 5 min, 6 h, and 24 h after running 63 km. *Denotes *p* < 0.01

For the plasma biomarkers, one participant was below the detection limit for hs-cTnT of 3 ng/L and six participants were below the detection limit for haptoglobin 0.2 g L^−1^ at one or more time points. Mean concentrations of novel biomarkers MR-proANP and copeptin-us showed marked changes at < 5 min post-race compared to pre-race concentrations (*p* < 0.001), and the latter was also significantly different from the pre-race concentration at 6 h and 24 h post-race (*p* < 0.001) as was hs-cTnT (Fig. 3). Several other biomarkers showed mean post-race concentrations significantly different from the pre-race concentrations including at 24 h post-race (Table 3).

LVEF and LV GLS were significantly associated (ß = − 0.8, within-individual *R* ^2^ = 0.18, *p* < 0.02). LVEF was significantly associated with copeptin-us (ß = − 0.8, within-individual *R* ^2^ = 0.30, *p* < 0.039), hs-cTnT (ß = − 2.9, within-individual *R* ^2^ = 0.36, *p* < 0.001), but no statistically significant age-adjusted correlations were found between neither copeptin-us nor hs-cTnT with RV GLS or LV GLS. Of these biomarker and echocardiography measurements, only LVEF was significantly correlated with estimated VO_2_max (ß = − 0.34, *p* < 0.007), while none of these parameters were significantly correlated with duration of race time (data not shown). Pre-race LVEF could not explain the post-race LVEF development (*p* = 0.734), and no interaction between VO_2_max group (< 52.5 and > 52.5 estimated VO_2_max) and post-race LVEF measurement was found (*p* = 0.854), and the inverse relationship between LVEF and VO_2_max was no longer statistically significant (*p* = 0.096). When presented separately in a similar multivariate regression analysis (co-variables: post-race LVEF, pre-race LVEF, and age) for the two VO_2_max groups, the post-race time points showed similar albeit non-significant results, i.e., high VO_2_max (ß = 0.12, *p* = 0.150) and low VO_2_max (ß = 0.17, *p* = 0.150). Race time and VO_2_max were inversely correlated (ß = − 1.40, *p* < 0.001).

The HR-corrected QT interval at 24 h post-race increased by 28 ms (*p* < 0.011). Other time intervals did not change significantly post-race (Table 3).

## Discussion

Several functional cardiac measurements as well as plasma biomarkers showed that the participants of this study had acute and sub-acute exercise-induced impact on cardiac function. However, it is of note that several cardiac and kidney biomarkers remained elevated at 24 h post-race. Furthermore, running intensity was relatively high at 72% VO_2_max.

For cardiac output (increase) and LV end-diastolic volume (decrease), the values were only altered immediately post-race. As expected, cardiac output increase was due to an increase in HR but independent of a significant decrease in stroke volume. LVEF functionality decreased immediately and at 6 h post-race, and VO_2_max was not significantly associated with the decreased post-race LVEF in contrast to other studies [26]. A possible mechanism to this feature relates to the volume overload of LV presuming that it is greater with higher VO_2_max. Initially, the preload is increased with almost maximal use of the Frank-Starling mechanism with some increase in systolic aortic pressure. The ejection fraction would remain the same as these two changes annul each other. Thereafter, the left ventricle dilates and becomes hypertrophic resulting in chronic overload which produces further elongation of myocardial fibers. Eventually, the cardiac muscle fails to maintain a match between pre- and afterload following which ejection fraction declines [26]. The possible mechanism just described refers to LVEF during exercise, but hardly remains a factor in the acute phase following cessation of exercise, i.e., until 30 min post-exercise when the first post-race echocardiographic scanning was carried out. Nevertheless, there could be other regulatory mechanisms which may have contributed to the decreases in LV function, including also the sub-acute post-race decrease in LV end-diastolic volume. These include a down-regulation of cardiac β-receptors mediated by elevated catecholamines during endurance exercise [27, 28] or alterations in myofilament sensitivity to calcium [29] among others. It is of note that the decrease in LVEF in the presence of decreased systolic blood pressure which remained significant up to 24 h post-race reflects depressed cardiac contractility. In combination, the decrease in LVEF and LV end-diastolic volume can be interpreted as “cardiac fatigue” [30] which is a common feature following endurance events as demonstrated in several other studies focusing on prolonged exercise of different distances [31, 32]. Furthermore, in spite of the non-significant changes in LV GLS following the ultra-distance race, the changes in LV GLS and LVEF were significantly associated. There may have been a lack of statistical power to show a post-race within-individual change in cardiac strain despite the acute change in preload.

Plasma copeptin-us—a stable C-terminal portion of arginine vasopressin precursor—was inversely associated with post-race LVEF. As a marker of dehydration and hypoxia, elevated plasma copeptin-us indicates that post-race LVEF could partly be explained by dehydration as well as a state of hypoxia. Evidence for dehydration was also supported by the decrease in mean LV end-diastolic volume 30 min post-race and by mean velocity time integral which was decreased up to 6 h post-race. Furthermore, the significant increase in mean plasma albumin at < 5 min post-race by 4.5 g L^−1^ is strong evidence for a state of dehydration.

Hypoxia may also be assessed by plasma hs-cTnT which showed an inverse association with LVEF; this further suggests a state of cardiac hypoxia as a consequence of the ultra-marathon race performed under extreme altitude-changing conditions. This is in contrast to earlier studies which did not find any associations between changes in LVEF and post-race cardiac troponins [2, 5] despite an increase in cardiac troponins in various ultra-exercise events lasting up to ~ 30 h of duration and one [5] being a 100 miles foot race covering altitude differences comparable to the current study. A likely explanation for this discrepancy is a difference in exercise intensity and/or altitude change during exercise with 1800 m of especially uphill running in the present study as a major influence on cardiac strain and functionality.

It is of note that except for mean RV S’ which decreased by ~ 13% 30 min post-race, none of the RV functional measurements were significantly altered post-race. Previous reports did, however, show substantial exercise-induced RV dysfunction in endurance athletes [2, 15, 33, 34], and in some cases, RV dysfunction persisted up to 1 month following an endurance exercise event [34].

Based on the absolute as well as relative intensity (9.1 km h^−1^ and 72%, respectively), the athletes in our study did race at a the lower end of high intensity (≥ 70%) which is a little higher than we had expected based on previous reports [7] but also based on the estimated VO_2_max of 54.5 mLO_2_·min^−1^·kg^−1^. The latter indicates that the intensity of their regular exercise pattern would be of moderate intensity as reported VO_2_max were similar (~ 58 mLO_2_ min^−1^ kg^−1^) in other amateur ultra-endurance athletes [3]. It is noteworthy that Neilan et al. [35] demonstrated that the majority of the most marked cardiac structure and function as well as cardiac biomarkers were observed in athletes training less than 35 miles week^−1^ before a marathon race, i.e., at high intensity. Male Tarahumara in general have most likely had life-long exposure to ultra-distance running at low to moderate intensity suggesting that exercise intensity may be more important in acute and chronic RV dysfunction than exercise duration per se. Nevertheless, it is important to add that combined age and sub-maximal derived VO_2_max has an uncertainty of ± 12 O_2_ min^−1^ kg^−1^ [22, 36] making a validation of the 8-min step test high priority for the sake of interpretation.

In support of an exacerbated strain on the cardiac muscle when competition is conducted in terrain of altitude variation with descent and ascent compared to no altitude variation was our finding on plasma MR-proANP. It remained significantly elevated in the present study at 24 h post-race. However, in a study we conducted in a group of male Tarahumara running 78 km on a flat route at 2400 meters above sea level and carried out at similar absolute but slightly lower relative intensity compared to the present study, post-race elevated MR-proANP was cleared at 1 h post-race [7].

Renal function was also compromised as a result of the physical exertion the runners underwent. Apart from plasma copeptin-us—which had increased ~ tenfold immediately post-race and remained significantly elevated at 6 h post-race—plasma creatinine also remained elevated at the 6 h post-race time point with an increase of ~ 35%. These results confirm a post-race increase in plasma copeptin-us and creatinine similar to what we reported from an earlier ultra-distance race in male Tarahumara [7] as well as shown in several other studies based on distance races ranging from 42 to 100 km [1, 15, 37]. Likewise, copeptin-us and creatinine increase could have been caused by dehydration. Furthermore, one participant reported having previously experienced hematuria following a race which suggests that at least some of the Tarahumara runners acutely overexert kidney functionality.

Several other plasma biomarkers remained significantly elevated at 24 h post-race including CK (~ 40-fold increase) CK-MB (~ 20-fold increase) and LDH (~ threefold increase) which indicates severe skeletal muscular damage that could have been exacerbated by eccentric muscle contractions when going downhill. This is supported by laboratory-based results of increased serum CK up to 48 h following 45 min of downhill running [38].

It is of note that one participant had elevated blood pressure at pre-race measurement. However, without follow-up measurements in a clinic we would be reluctant to define the participant as having hypertension. Furthermore, “white coat hypertension” caused by apprehension is a likely cause of the elevated blood pressure. Furthermore, another participant was anemic, which is a common trait in individuals living under constrained dietary conditions like the Tarahumara. It is likely that the anemic state negatively affected the performance of this individual and thereby the results of the echocardiographic and biomarker measurements.

We acknowledge several limitations to this study. In spite of an attempt to include a non-Tarahumara control group participating in the same race, we did not succeed primarily due to the time line with the 24-h follow-up measurements. We did not obtain post-race body weight measures as an indication of dehydration in spite of the useful information from a variation of this parameter [39]. Instead, we used albumin as a marker of dehydration and adjusted all plasma biomarker analyses for albumin in order to present biomarker results not confounded by dehydration. VO_2_max was estimated using the HR as a proxy for oxygen uptake based on the linear relationship between VO_2_-uptake and HR. Furthermore, the protocol was sub-maximal using predicted maximal HR according to Tanaka et al. [36] for the assessment of VO_2_max. This indirect methodology may have resulted in a ± 12% deviation compared to an indirect calorimetric measurement. Lastly, the low number of study participants (*n* = 10) may have introduced a statistical type II error, and the generalizability to other Tarahumara must therefore be done with caution.

## Conclusion

Following a 63-km race with extreme altitude variation run at high intensity, the Tarahumara athletes participating in this study showed acute, transient cardiac and kidney dysfunction as assessed by echocardiography and plasma biomarkers. LV dysfunction was more apparent than RV dysfunction, but both were normalized < 24 h, while several plasma biomarkers remained elevated at 24 h post-race. The lack of transient RV dysfunction warrants further studies with longer running exposure in order to investigate a possible threshold for compromising RV function in this population and possible explanatory mechanisms. In addition, possible permanent cardiac dysfunction following life-long exposure to ultra-distance running as practiced among the Mexican Tarahumara should be studied with a non-Tarahumara reference group who have taken up ultra-distance running in adulthood; this way the life-course duration of ultra-distance running as an exposure factor for cardiac dysfunction can be elucidated and thereby further qualify medical advice for ultra-endurance athletes.

## Additional file

**Additional file 1.** Physical activity questionnaire.

## Abbreviations

copeptin-us: copeptin-ultra sensitive
CK: creatine kinase
CK-MB: creatine kinase isoform myocardial band
ECG: electrocardiography
HbA1c: glycated hemoglobin
Hb: hemoglobin
HR: heart rate
HRmax: maximum heart rate
hs-cTnT: high-sensitivity cardiac troponin T
LDH: lactate dehydrogenase
LV: left ventricular
LVEF: left ventricular ejection fraction
LV GLS: left ventricular global longitudinal strain
RV: right ventricular
MR-proANP: mid-regional pro-atrial natriuretic peptide
VO_2_max: maximum oxygen uptake
