# Supplementary material for: Transient cardiac dysfunction but elevated cardiac and kidney biomarkers 24 h following an ultra-distance running event in Mexican Tarahumara
Source: Extrem Physiol Med. 2017 Dec 11;6:3. doi: 10.1186/s13728-017-0057-5 (PMC5725886; doi:10.1186/s13728-017-0057-5)
Supplement: Supplementary file 1 — Additional file 1. Physical activity questionnaire. [file 13728_2017_57_MOESM1_ESM.pdf]

Physical activity questionnaire Guachochi, Chihuahua, México, July 2012

Study no.________________________ Sex__________

Name____

Born (place and country)____

Date of birth (day/month/year)_______________________________________ Age________

Country of citizenship_____________________ Country of residency__________________

Origin of father__________________________ Origin of mother______________________

Origin of paternal grandfather ______________ Origin of paternal grandmother__________

Origin of maternal grandfather______________ Origin of maternal grandmother__________

1. When did you start running (organized training/competition)_________________________________

1. Desde cuando comenzó usted a correr para competir (entrenamientos y competencias)?

2. Did you walk/run to and from school?___________________

2. Cuando usted iba a la escuela o a otros lugares, se iba corriendo o caminaba de un lugar a otro?

3. If yes, for how many years and how far____

3. Si corría o se iba caminando de un lugar a otro, por cuantos años lo hizo?

4. How long of a distance did you use to run on those occasions, and how many days per week, more or less?____

4. Qué distancias recorría usted en esas ocasiones y cuántos días por semana, más o menos?

5. (For non-Rarámuri runners): At what age did you start training for long distance running?_______________

5. (Para competidores no-Rarámuris): a qué edad comenzó usted a entrenar para carreras de gran distancia?

6. Did you ever have to stop your training program because of injury or disease?

Yes______ No_______

6. Has parado tu entrenamiento por causa de una lesión o enfermedad?

Sí_____ No_____

7. (For Raramuri only):If you used to cover those distances either running or walking, did you have to stop doing it for some time because of injury or disease?

Yes_____ No_____

7. (Para Rarámuri solamente) Si usted hacía sus recorridos caminando o corriendo, tuvo alguna vez que dejar de hacerlo por lastimarse o enfermarse?

Si________ No______

8. If yes, could you please specify the cause____

8 Si dice si, por favor especifica la causa

9. (Non-Rarámuri only): for how long could you not continue your training?______________________

9. Por cuánto tiempo no pudo usted continuar su entrenamiento?

9. (Para Rarámuri solamente): Cuál fue la razón por la que tuvo que dejar de correr?

10. When you had to stop running due to injury/illness, did you then engage in any other sport (f. ex. Swimming, bicycling)? ____

Yes______ No_______

10. Cuândo detuvo su entrenamiento debido a lesión/enfermedad, participó en otro deporte (por ejemplo, natación o ciclismo?

Si_______ No_______

11. Specify your running pattern over the past year?________________________________

____

11. (Para Rarámuri solamente): Puede describir lo que corrió (entrenamiento y carrera) desde el año pasado hasta ahorita?

11. (Para no-Rarámuri solamente) Podría describir brevemente su programa de trabajo (entrenamiento y competencias) durante el ultimo año?

12. How many competitions have your done over the past year?______________________________

12. (Para Rarámuri solamente) En cuántas carreras corrió usted desde el año pasado hasta ahorita?

12. (Para no-Rarámuri solamente)En cuántas competencias participó usted durante el ultimo año?

13. What is a typical competition distance?____

13. (Ambos grupos de competidores): Cuál es su distancia típica o habitual de competencia?

14. What is the furthest you have competed over the past year? Ever, in both training and actual competition?____

14. (Ambos grupos de competidores): cuál fue la carrera más larga en la que participó del año pasado para acá, tanto en su entrenamiento como en competencia? Cuál ha sido su distancia más larga en entrenamiento o carrera en toda su vida?

15. Have you ever experienced chest pain in connection with running? If yes, please specify!

____

15. Ha usted experimentado alguna vez dolor en el pecho que pudiera estar relacionado con correr? Si le ha ocurrido, podría usted dar más detalles?

16. Have you ever had blood in your urine, either after training or competition?________________

16. Ha usted orinado sangre después de una carrera o de un entrenamiento?

17. If yes, did you experience any symptoms? If yes, please specify_________________________

17. Si su respuesta es sí, ha experimentado algún síntoma? Por favor, especifiquelo
